# An Essay on Milksickness

**Published:** 1854-09

**Authors:** S. W. Thompson

**Affiliations:** Marshall, Ill.


### THE NORTH-WESTERN
### MEDICAL AND SURGICAL JOURNAL.

NEW SERIES.

VOL. III. SEPTEMBER, 1854. NO. 9

### ORIGINAL COMMUNICATIONS.

ART. I.—*An Essay on Milksickness.*—Read before the Æsculapian Medical Society, by S. W. Thompson, M. D., Marshall, Ill., May, 31st, 1854.

*Gentlemen:*—It is with peculiar diffidence that I attempt to lay before you an Essay at all worthy of your consideration, or the subject at the head of this Article, for I am, as every young practitioner must be, deficient in that all-important acquirement to a medical man, experience; but having been appointed one of your essayists, I will not be the first to shrink from any honorable responsibility or duty devolving upon me as a member of the noble profession we are bound to cherish and respect. At the present time it especially behoves each and every one of us to exert ourselves to the utmost for the advancement of ourselves and profession, in order by making both respected, to put down the host of empirics and nostrum venders who are ever hovering round, ready to pounce upon and destroy the poor victim to public credulity.

It is not my intention in this paper, even were I capable of so doing, to enter into a full and elaborate discussion of the subject of Milksickness, but merely to state the views held by myself and some others as to its cause; and then to express an opinion, or rather make a suggestion as to which species of disease it should be classed with. As regards the latter, I am well aware that I am at present unable to substantiate my theory by proof; for time must elapse, and investigations be entered in to, ere facts can be elicited sufficient to allow us an opportunity of arriving at any certainty regarding the peculiar pathological phenomena to be observed in the disease. Therefore nearly or quite all our arguments and reasoning relative to the cause and character of Milksickness are based upon *mere theory* and must not be allowed the same weight or influence upon our views, as if they were the result of actual observation or experience.

I shall therefore first briefly mention where milksickness is found or said to be endemic. Upon this point our information is very definite. We see the disease prevailing upon low bottom lands in the vicinity of water courses, or in low marshy prairie or their neighborhood. We occasionally also notice it in comparatively elevated table lands which are thickly covered by a growth of underbrush or young timber. But the places where we find it most frequently and where at the same time it assumes its most severe and grave type are on the bluffs overhanging creek or river bottoms. These bluffs have been chosen as building spots by the settlers on account of their being above high water mark, and also from an erroneous idea that high lands must be salubrious.

It is true we occasionally see the disease in situations other than those here described, but it is rare and must be looked upon rather as exceptional and sporadic, and not at all militating against the assertion that it usually prevails in and close to malarious localities. Almost all authorities agree upon this point. Dr. Drake would be still more definite, although he does not agree to the hypothesis of its being miasmatic in its origin. He says that the localities giving rise to it are only "the white elm slashes in post oak flats," but this very limited location is no longer tenable, and indeed it is strange that the father of western medicine should ever have taken up with the conviction which he here expresses. It would be a work of supererogation for me to give a list of districts in this portion of the state, where milksickness prevails; or of the authorities who have testified to the truth of its most frequent occurrence in such localities as I have described. You are doubtless fully acquainted with both, and I shall now proceed to consider some of the theories which have been advanced as to its cause. There are I believe but four of these which require special notice, viz.: the Animal, Vegetable, Mineral and Malarial.

The advocates of its Animal origin maintain that it is exclusively in man, derived from the use of beef, butter, milk or cheese obtained from an affected beast. In answer to this it is only necessary to prove that it *may* occur without the possibility of any of these articles being the cause. That such can be proven is very evident. A single instance quoted by myself in a paper published in the Western Journal of Medicine and Surgery for June, 1853 is sufficient to settle this point. In this instance every thing belonging to cattle, or obtained directly or indirectly from them, was for a space of about seven years entirely interdicted from being used in the family. But see the result. Was the family exempt from the disease? By no means; they suffered from it as severely every fall as previously That bad or unwholesome food of any kind may cause a *manifestation* of the disease I can readily believe. Analogy in other cases would forbid a denial, unless proof were present to give a coloring to my incredulity. But to say that the disease *could not* occur without such food being used; to assert that such articles as I have above enumerated, are the essential first and last causes of the malady, is what I cannot believe

The reasons given for a belief in this theory by its supporters, are numerous, and some of the arguments adduced are specious, and bear with them at a cursory glance the appearance of truth.— But upon a closer inspection we find them either incomplete or based upon an a priori mode of reasoning which will not bear analysis. For instance, it is asserted that dogs or other animals, after eating of the carcass of a beast dead of milksickness or trembles, are attacked with the same disease, or one supposed to be so and it probably is. But when we come to examine for ouselves, we find that the number so attacked is but a small ratio of those which have partaken of the diseased beast, and those attacked are not always the one taking the largest amount of the diseased flesh, but rather such as stay longest in close proximity to the noxious effluvia arising from the carcass; and as the beast usually dies in or close to the locality where the disease is supposed to originate, it is but fair to admit that the dog was subject to the same cause of disease as the cow. And again, if there is any peculiar essential poison in the dead cow, we should reasonably expect to see the usual manifestation of the poison, in those animals partaking thereof, and these effects should be most plainly exhibited in such as had taken the largest amount of matter containing it It is true that some men, and this fact applies equally to other animals, are more susceptible of a given amount of some agent than others, and this may possibly be the case in the present instance But Philosophy teaches us to search for no more reasons to explain a phenomenon than are sufficient to account for it, and here I maintain that exposure to the same atmosphere, or if you please, the same cause that gave rise to the disease in the cow, sufficiently explains the phenomenon of the dog being seized with a disease *supposed* to be the same. But a little farther. If the diseased meat, &c , causes it in man and dog, what gives rise to it every fall in the cow? Here we must look again and attempt some other solution of the difficulty, and I would ask, if there is some other efficient cause giving rise to it in the beast, whether this same may not give rise to it in man? The unprejudiced and reasonable mind at once suggests an affirmative answer. Again: It is affirmed that when flesh of a diseased beast is given to dogs not allowed to go to the carcass, it has caused milksickness in them. Such may be the fact, but in the absence of *some* of the facts connected with the case, and moreover in consideration of these phenomena having *apparently* been observed by men incapable of giving them their due weight and value, and of drawing just deductions from such facts as *were* observed, whilst they were liable to be misled by preconceived prejudices, we must be very cautious how far we allow such statements to be received.

Let us now take a brief survey of the claims which the theory of the vegetable origin of the disease presents to our notice.—

This theory has had and still has many advocates, amongst whom is I believe Prof. Dickson of S. Carolina. The late Dr. Drake was also amongst its supporters. There is a peculiarity about this view of the question which is rather singular, considering the certainty which has been expressed, by those writing in favor of this theory, in regard to the special cause. It is this: that there are scarcely any two who agree upon the particular vegetable product, which they would persuade us is the first and only cause of the disease in question. The Eupatorium Ageratoides, Bignona Capreolata, Rhus Toxicodendron and some of the Fungi have been cited as specific causes of milksickness. Besides those here enumerated there are a host of others too numerous to mention and unworthy of notice. Dr. Drake was led to believe the Rhus Toxicodendron was the noxious vegetable, but when we come to examine the subject we find not a shadow of evidence to substantiate his theory. Indeed the chief ground upon which the vegetable theory is based, are "that each writer has found some *one* of these supposed noxious vegetables in the immediate vicinity of where the disease is supposed to originate, and in some cases they are said to have eaten of them, and been subsequently attacked with the disease" Ergo, the plant gave rise to the malady.

There are one or two facts that I will mention in opposition to the above. In the first place, nature has wisely endowed her creatures with instinct sufficient to lead them to pluck only such food as is suited to their wants, and to shun such as is deleterious. This fact is too well known to need confirmation, and although domesticated animals sometimes partially lose this instinct, yet it could not be the case with cattle, used to getting their entire sustenance during the spring, summer and fall from the unenclosed prairie and wood lands. Secondly, were this theory correct, we should find the disease only in such places as this vegetable, whichever one it may be, and is likewise discoverable. But is this the case? By no means, for when we find some of them to exist, there are not others. This question might forever be set at rest by separating a number of cattle and feeding them on the various vegetables supposed to be hurtful, at the same time they are free from any of the other supposed causes of the disease. I cannot pass this part of the subject over without a cursory notice of a new causus morbi which has lately been discovered. or at least said to have been so; and coming as it does from a high source, it might from that cause alone have more influence than it deserves. The substance to which I allude is a kind of fungus similar to the secale cornutum. and its effects are claimed to have been discovered by Prof. Slack of the Cincinnati College of Medicine and Surgery. The claims of this article to the *enviable notoriety* of being the cause of so much ill to nature's animated creation, are warmly advocated by this gentleman in a paper published in the Western Lancet for March, 1854. This paper is headed ' Ergodeleteria," and is plentifully interspersed with quotations and italicising. The learned professor speaks in term of derision of the various theories hitherto advanced, and then proceeds to declare in a very positive manner, the certainties which accompany his solution of the much vexed question of the cause of milksickness — Speaking of all previous theories he says "to the writer of the present Essay, all these particulars are but *guesses*, most of them fanciful and fall quite short of the *truth*." And what after all are his reasons for presuming he has discovered the true cause. and thus condemning all previous productions "as mere guesses and but fanciful?" Simply these. if grounds indeed they are. That in all the Graminaciæ or grass tribes, the place of the seeds is sometimes occupied by a *morbid growth*. That this abnormal production is similar in its nature to the ergot of rye, which from ample experience has been proven capable of generating disease in both man and beast. He then goes on to state that "early in November of 1839 or '40, by request I visited a very respectable family. I found them all laid down with *milksickness*. The house was a hospital—all were sick. The lady was retching and vomiting in a manner *unusual to me*. She died during the night after.' He then gives a description of the land and what was found there. "The family lived in a fertile place, one and a half miles from Memphis, Tenn., on the east bank of Wolf river. The whole place except a small strip of land, was frequently overflowed by water. In this particular case I looked around carefully for a cause of the very distressing malady, and was satisfied it re[illegible]ed from something produced in the vigorous grasses of these *low lands*. since which botanical facts confirmed my impressions, and led to the sentiments above. There is an inconsistency in the professor's descriptions, which I could wish he had avoided. We might suppose this was the first attack of the kind he had ever seen, and not knowing what else to call it, gave it the accommodating name of *milksickness*, for he says he found the woman "retching and vomiting in a way unusual to him." If he was acquainted with the disease either by experience or otherwise, this could not have been the case, for when a physician has seen one or two patients vomiting in this complaint, the scene is not easily effaced from his memory, and this symptom is usually particularly and minutely described by those who have written upon the disease. But now the woman was dead, Prof. Slack with praiseworthy zeal seeks for the cause of disease, but at the time discovers nothing, if we except some rank vegetation, consisting mostly of grasses in the overflowed lands. *There may have been* the *blight* which he supposes to be the noxious article, yet he does not say it was found at this time. However, after "thirteen years" have passed he discovers that the "Ergodeleteria" was there.

This is a quasi medical philosophy which I neither know, nor wish to know anything of. Could not the occurrence of the disease been more rationally accounted for by supposing malaria to have been the cause. Here were all the elements necessary to its production, in their most concentrated form. Such of the symptoms as are described were certainly more like those present in some diseases acknowledged to be miasmatic in their origin, than those presented by poisoning from the secale cornutum. But this explanation would not have given Prof. Slack the opportunity and eclat of promulgating a new theory, no matter how absurd soever it might be. But after all, the gentleman is no nearer the solution of the difficulty than at first, for his theory would only account for its occurrence in the beast, and unless we suppose that this "Ergodeleteria" remains in a sufficiently concentrated form to poison the meat to an extent fatal to all partaking thereof, and also the butter and milk during life, we have no solution at all. The theory of its mineral origin, with such a supposition as this to assist it would be far more worthy of credence.

In considering this latter theory of the origin of milksickness I shall be as brief as possible. It is supposed that in the fall of the year, when the waters are low, there is some noxious mineral suspended in them, and the cattle when they go to drink at the creeks and pools, receive this poison with the water and thus contract the disease. There are several mineral productions which have been supposed to exert this baneful influence, but what the grounds for a belief in this view of its etiology are, I cannot conceive, nor will this theory bear the test of investigation any better than those we have just considered. A remark which I previously made in reference to its vegetable origin, applies equally against the theory now under consideration; for whatever mineral we attribute the disease to, such should be found constantly present in the waters which are said to give rise to the disease, and when such is not the case we should not expect to find the effects of a poison which does not exist. Dr. Drake in his memoir on milksickness, denies there having been any one mineral product constantly found in the water of the neighborhoods supposed to give rise to this malady. There is a slightly modified view of this question taken by some of the advocates for its mineral etiology. This is, that the poisonous article, whatever it is, comes up in the form of vapor, and is deposited upon the vegetation, and thus taken by the cow. But this view might be made to assist any other theory as well as this, and experiments so far, have not proved any thing capable of generating this disease, to be so deposited.

I have now briefly considered *three* of the *four* most commonly received opinions of the etiology of milksickness. I shall therefore now proceed to examine the remaining *one*, which I believe is the most rational, philosophical, and in best accordance with our actual information relative to the disease.

The best way to examine this theory, will be first to give some of the reasons assigned for its support, and then examine these reasons to see whether they are valid and apparently substantiated by facts. To proceed then; it is maintained first, that milksickness usually if not invariably occurs in malarious localities, or such as are subject to miasmatic influences.

Secondly, that the disease in many instances at least, can be proven to have occured without any of the causes assigned by the advocates of other theories being present, and therefore in want of better evidence we are justified in attributing it to malaria.

Thirdly, its resemblance to certain forms of disease which are almost universally conceded to be caused by malarial or miasmatic influences.

Such are some of the reasons assigned by the advocates of this theory for a belief in its cor ectness. I will now proceed to consider these reasons *seriatim*.

First, That milksickness usually occurs in districts malarious in their character, is as I have before observed, almost universally admitted. Its most frequent habitation is in places where Remitent, Intermittent and Congestive fevers of various grades are most frequently and constantly seen. That we do occasionally see the disease in situations where this cause does not appear very manifest, cannot be denied, but then we do not know to what extent a patient attacked with milksickness, may have been exposed to malarial influences in another place, perhaps days before and entirely forgotten by himself. Further; we know that currents of air or other causes sometimes extend the influence of miasmatic or malarial vapors to an indefinite extent, and in such cases, I have little doubt, that could the chemist be upon the spot at the time, and were he possessed of the means of analysis sufficiently nice, the presence of the malarial vapor could be demonstrated.

Secondly, of the occurrence of the disease independent of the so called causes assigned by the supporters of other theories. This can be proven beyond a doubt. Families have been attacked, who have most rigidly abstained from the use of every thing which could give rise to the disease, through the medium of the beast either dead or alive; as see the case referred to in the previous part of this paper. In this instance the same water was used all the year through, and yet the disease never showed itself until the latter part of summer or fall when the decay of vegetation commenced. This family however lived in the immediate vicinity of a swamp, at all times wet, and sometimes overflowed to a depth of several feet. There was likewise a flat swampy piece of prairie, extending up close to the house. This prairie was always covered with a luxuriant growth of vegetation, mostly of a succulent character. At the suggestion of the family physician, this low piece of ground was cleared and drained, thus getting rid of a great portion of the superabundant moisture, and exposing the soil to the influence of the suns rays. What was the consequence? The family were afterwards, almost exempt from the disease, and only suffered when the swamps adjoining was repeatedly overflowed during the summer.

We frequently see instances of a family remaining entirely exempt, until some low rich piece of ground, upon which a thick layer of dead leaves and grass has remained undisturbed for years, is broken up, and the only partially decomposed vegetation becoming exposed, a fresh decomposition takes place and the family is then troubled with milksickness for several years, or until the previous accumulation of dead leaves &c., are entirely metamorphosed into productive and innoxious soil.

Again: cattle have been attacked by the disease when grazing in a low piece of meadow land, where they had no chance of getting water other than from the same well that the family used from; and why should a poisonous vegetable now spring up and be eaten by the beast, when it had never made its appearance previously? In these as indeed in other cases, the family as well as the beast remained entirely exempt until fall or early winter, and in many instances where the *family lived* upon an elevated and salubrious situation, *they* remained free of the disease for the entire year.

Thirdly, its resemblance to certain forms of disease, generally acknowledged to be miasmatic in their origin! This is a point which so far as I am aware, has never before been advanced in an essay on milksickness, but I am firmly convinced that the resemblance does actually exist, that I have not based my belief upon unreasonable premises.

It is generally conceded, that in diseases spoken of as *congestive* in their character, the first morbid impression is received upon the *nervous system*, speaking of this in its most comprehensive signification. That is to say, that the morbid cause, be it what it may, first exerts itself upon the nervous filaments. For instance, one of the first steps in an inflamation, is congestion. But prior to the existence of the engorged condition of the vessels, essential to this state, there must be, and undoubtedly is an impression made upon the nervous filaments supplying the parts to which these vessels are distributed, and the vessels themselves; this impression results first, in a loss of innervation, or in other words a loss by the nerves of the "*vis conservatrix natura*" by which she throws off any temporary disposition to diseased, or abnormal action. In congestive fever we believe the peculiar pathological phenomenon, whence the disease derives its name, is induced by an impression made upon the nervous system, resulting in a want of power or or loss of innervation, which in its turn causes a condition of partial stagnation or congestion upon the internal organs. We know that a deficient supply of nervous influence causes coldness or a loss of the normal temperature of the parts where such deficiency exists, as in paralysis. So in congestive fever; the first impression being made directly or indirectly upon the nervous centres, the afflux of blood is towards the internal organs, because the distal extremities of the nerves are the ones upon which falls the weight of the morbid cause, their healthy action depending upon an integrity of the central organs whence they spring. The congestion thus resulting may in its turn terminate in local inflammation, such of course occurring in the weakest organs, and the ones most prone to take on this state.*

It will be inferred from the preceding remarks that it is to the congestive variety of disease that milksickness belongs; or rather that it is but a variety of congestive fever.

Let us now examine the symptoms, and see if they correspond with, or can be accounted for by this theory, and also whether they are, or are not analogous to those present in the varieties of congestive disease to which I have more special reference.

First then of the *pulse*. This find to be weak, but usually not much increased or reduced in frequency, or irregular in its beats.—It is rather what would be called a pulse of depression, or one which from sympathy with the reduced powers of the system generally, lacked its accustomed vigor. The heart however appears to beat with great violence, as if clogged and obstructed in its ac-

* "See Fordyce on fever."

tion, but in spite of its apparent strength it does not propel the vital fluid with its accustomed force. It is the beat of weakness —that of the organ attempting beyond its power to throw off the accumulation of blood which obstructs the normal action, and the discharge of its accustomed functions. We find the skin cool, or cold, without the patient complaining of such being the case. This we should naturally expect, if there is, as I suppose, a lowered condition of the vital functions, owing to deficient innervation.

The capillary vessels of the skin, having a deficient supply of nervous influence, are, from this cause, and the mechanical contraction of their parieties, resulting from reduced temperature following such deficiency, likewise deficient in their accustomed supply of blood. Restlessness and trembling are also very constant symptoms. These we find present in almost any disease in which the powers of life are depressed, or reduced to an inordinate extent. We often see them when a person is weakened by the loss of blood or great fatigue. They are but evidences of reduced power of the whole economy.

Again—the obstinate constipation of the bowels is not, in my opinion, the result of any structural derangement, but rather of a deficiency in the accustomed peristaltic and contractile efforts of the intestines. I am by no means satisfied that it is not a condition similar, and dependent upon a somewhat similar cause as is this symptom in colica pictonum; and, so far as we have pathological appearances to guide us, we are justified in the opinion. In the few *post mortems* which have been made, and of which we have reliable accounts, there have been no signs of actual inflammation in the intestines, although there is said to have been some slight abnormal redness, dependent, perhaps, upon retention of the hardened feces within the delicate lining of the bowels, or else caused by the stimulating and irritating medicines so commonly administered, for we are told that this slight redness is chiefly limited to the upper portion of the alimentary canal, although not entirely. Nausea and vomiting are also very constantly present; yet I see no reason why we may not explain their occurrence in a manner entirely consistent with my theory of the disease. What is more distressing than the nausea and vomiting sometimes attending pregnancy, or in some varieties of dyspepsia? Yet we find in these cases no organic change. They are the result of functional derangement merely. Yet, in the latter affection, the sense of gnawing and burning at the epigastrium is almost, or quite equal to the same symptoms in milksickness—indeed the appearances after death presented by the stomach are not dissimilar. In milksickness, it is true, this organ appears more red, either the result of *recent* inflammation or excessive congestion. It is not unlikely that in protracted cases, which prove fatal, the constant and severe retching and straining may have a tendency to cause the existing congestion to progress to a state of inflammation: but we must recollect how apt the inexperienced student in pathology is to mistake mere congestion for inflammation.

It may be objected that the term "loss of innervation" is vague, and in itself without meaning. This is true; but, in the present state of our science, we are obliged oftentimes to use terms the strict interpretation of which we are not acquainted with; but they, nevertheless, serve to convey an idea of the condition of disease, the cause and complication of which we do not rightly understand.

*I then believe that there is no* PECULIAR *disease known as milksickness*, but that the disease so called is neither more nor less than a variety of our congestive fever, of course, modified by such circumstances as constitutional or temporary peculiarities of the patient; difference in the nature or virulence of the miasmatic or malarial poison, and also somewhat by the season at which the disease usually presents itself. I have previously expressed the opinion that the primary impression is made upon the nervous system. Whether it is upon the animal or organic system chiefly, I cannot say; but, from many circumstances attending the disease, I think it probable the latter is the one chiefly implicated. For instance, the secretory and circulatory systems each show great evidences of derangement. The natural temperature of the body is reduced; the functional derangement of the stomach and bowels without sufficient organic lesion to account for it, would all lead us to adopt this conclusion, as these various processes and organs are more particularly under the control of the great sympathetic system; but, from the close sympathy and connexion existing between the two, it is not probable that either could be greatly deranged, and the other remain intact.

The first impression of the disease *may be* upon the blood, through the medium of the respiratory apparatus; but of this we have no data from which to judge, and all our opinions upon this point are mere hypotheses.

We here find, then, that the symptoms of congestive fever and milksickness are not unlike; that in many respects they are actually identical The particular grade of congestive attack to which I consider it to bear the closest resemblance is, a combination of the algid and gastro-enteric varieties. Although the symptoms are not precisely similar to either one variety, yet in cases where we find an union or blending of these two varieties, we not unfrequently see a combination of symptoms almost, if not quite, identical with those here spoken of. That such a blending may take place is well known. Bartlett, at the end of his description of the symptoms of the several varieties of congestive fever, expressly says, "that two or more of these varieties may be united—sometimes the symptoms of one predominating, and sometimes those of another."

I am inclined to believe we are all a little too careless in our diagnosis of these attacks. If we see a well-marked case of congestive fever, we give it the proper name; but when we come to an array of symptoms similar to those seen in the disease called milksickness, we apply to them the popular name, because we know that these symptoms are present in the attack so called, and we fail to exert our analytical and reasoning faculties in endeavoring to class the disease in its proper place, but remain satisfied with telling ourselves "it is a case of milksickness;" and, this first wrong step being made, we are apt to commit another by treating for the *name*, and not for the *symptoms*. I am in hopes, if we all take proper pains, new and interesting discoveries will be made, which may enable us to see and treat this much feared disease, this bugbear of Western emigrants, with the same certainty and rationality as many of the best established in our nosological list.

I might, perhaps, have carried the analogy between milksickness and our common congestive attacks to a greater length with some benefit, but I conceive it to be unnecessary. The obstinate constipation is not usually present in the gastro-enteric varieties of congestive fever; but it is, to a greater or less extent, in the algid; and here I would remark, that when this symptom is overcome in milksickness, we not unfrequently see it followed by a transient diarrhœa, as also by some slight febrile movement.

Before closing, I must say a few words relative to the treatment of milksickness; and in this, I doubt not, I shall differ from most of you here present; but I cannot proceed without remarking how very desirable it would be to have some minute accounts of the appearances after death presented by the different organs of the body. Our information upon this point is most meagre and unsatisfactory. We know very little of the actual condition of the stomach and intestinal canal—nothing of the cerebro-spinal or great sympathetic systems of nerves. The latter remark applies equally to our knowledge of the lungs, liver, heart, &c. With such slender foundation, then, for our opinions of its nature, it is not unnatural we should fall into many erroneous hypotheses reregarding its actual character and treatment. I may have fallen into some of these; but, if I have, I will not be the last to discard my present views for such as are better supported.

You will, no doubt, expect, from the opinions I have expressed of the pathology of milksickness, that I should treat it in accordance with such views; nor will you be mistaken. But, from the peculiarly irritable condition of the stomach, we are debarred using remedies of an irritating or nauseous character until such irritability is allayed, and even then to any great extent, for fear of causing a return of the same.

The treatment I have found most successful consists, first, in the administration of

Hydrarg. sub. chlor. gr ij. vel.iij.
Soda bi carb. gr.v.

Repeated every one, two or three hours.

The soda bi carbon. serves to neutralize the excess of acid in the stomach, and the combination is almost invariably retained, and rarely fails in producing the effect desired. If this should not succeed, we may give the aqua camphora or small doses of morphia, or a weak solution of kreosote in water. I once tried this latter remedy in a case where all others had failed, and it answered the purpose intended in a most satisfactory manner.

Having now somewhat quieted the stomach, I give a brisk cathartic. This is beneficial in two ways: first, it stimulates the intestinal canal to its accustomed functions; and secondly, it is absolutely essential to evacuate the bowels of all offensive matters, or else the retching and vomiting are sure to return. I usually prefer the infusion of senna, or the pulv. jalapæ comp., because they are generally retained by the patient, and act in an efficient manner; but others may be used to suit the preferences of the physician, or to meet the exigencies of the case.

The excretory processes of the body must be renewed: and in no way can we do this with the same certainty as by evacuating the alimentary canal. If the remedies previously advised do not succeed, we have no time to lose.

The croton tiglii may be resorted to now with benefit, and its administration persevered in till an evacuation is procured, or the stomach becomes irritated by it. As soon as I have procured a full discharge from the bowels—and this we usually find to be of a tarry consistence, and very fetid—I begin at once the administration of quinine in some form. This I would have done previously, but, owing to the extreme gastric irritation, kept up, perhaps, by the retention of excrementitious matters in the intestinal tube, a remedy of so nauseous a character could not be retained.

The administration of this remedy is often followed by a tranquil sleep, and a warm perspiration breaks out all over the body. I continue the use of quinine for several days, or until the patient is convalescent. The tinct. ferri chlor., or some other preparation of iron, may now be substituted. You will at once perceive I have said nothing of general or local blood-letting or counter-irritants. The first I consider entirely inadmissible. The second in the shape of cups or leeches, if the latter can be had, to the epigastrium, I conceive *might* be beneficial, although I prefer counter-irritation by blisters or sinapisms, which I have usually found to answer every indication to be expected from their use. Sinapisms along the spine are also of benefit.

The general administration of irritating stimulants, such as brandy and whisky, &c., I do not approve of, as they can, in the inordinate quantities usually allowed, but serve to increase the gastric irritation already existing. Under certain conditions, however, weak brandy toddy or small quantities of wine are useful. I had forgotton to state that when I give cal. and sod. carb. the first dose or two is followed by a powder of cal. gr. x., chlor. sodium gr. x. This usually greatly assists the purgative subsequently administered in its desired effect.

I should think that the ferri et quinia citras would be a most excellent substitute for the quinine alone, being less bulky and nauseous, at the same time it combines the tonic and antiperiodic properties of both quinine and iron. I have never been able to procure it, but shall endeavor to do so, in order to test its efficacy in the disease now under consideration.

